# CD13 promotes mesenchymal stem cell-mediated regeneration of ischemic muscle

**DOI:** 10.3389/fphys.2013.00402

**Published:** 2014-01-09

**Authors:** M. Mamunur Rahman, Jaganathan Subramani, Mallika Ghosh, Jiyeon K. Denninger, Kotaro Takeda, Guo-Hua Fong, Morgan E. Carlson, Linda H. Shapiro

**Affiliations:** ^1^Center for Vascular Biology, University of Connecticut Health CenterFarmington, CT, USA; ^2^Department of Anesthesiology, Texas Tech University Health Sciences CenterLubbock, TX, USA; ^3^Center on Aging, University of Connecticut Health CenterFarmington, CT, USA; ^4^Drug Discovery, Genomics Institute of the Novartis Research FoundationSan Diego, CA, USA

**Keywords:** CD13, mesenchymal stem cells, adhesion, cell transplantation, hindlimb ischemia

## Abstract

Mesenchymal stem cells (MSCs) are multipotent, tissue-resident cells that can facilitate tissue regeneration and thus, show great promise as potential therapeutic agents. Functional MSCs have been isolated and characterized from a wide array of adult tissues and are universally identified by the shared expression of a core panel of MSCs markers. One of these markers is the multifunctional cell surface peptidase CD13 that has been shown to be expressed on human and murine MSCs from many tissues. To investigate whether this universal expression indicates a functional role for CD13 in MSC biology we isolated, expanded and characterized MSCs from bone marrow of wild type (WT) and CD13^KO^ mice. Characterization of these cells demonstrated that both WT and CD13^KO^ MSCs expressed the full complement of MSC markers (CD29, CD44, CD49e, CD105, Sca1), showed comparable proliferation rates and were capable of differentiating toward the adipogenic and osteogenic lineages. However, MSCs lacking CD13 were unable to differentiate into vascular cells, consistent with our previous characterization of CD13 as an angiogenic regulator. Compared to WT MSCs, adhesion and migration on various extracellular matrices of CD13^KO^ MSCs were significantly impaired, which correlated with decreased phospho-FAK levels and cytoskeletal alterations. Crosslinking human MSCs with activating CD13 antibodies increased cell adhesion to endothelial monolayers and induced FAK activation in a time dependent manner. In agreement with these *in vitro* data, intramuscular injection of CD13^KO^ MSCs in a model of severe ischemic limb injury resulted in significantly poorer perfusion, decreased ambulation, increased necrosis and impaired vascularization compared to those receiving WT MSCs. This study suggests that CD13 regulates FAK activation to promote MSC adhesion and migration, thus, contributing to MSC-mediated tissue repair. CD13 may present a viable target to enhance the efficacy of mesenchymal stem cell therapies.

## Introduction

Stem cells have the amazing capacity to contribute to the growth and healing of many different types of tissues and hold tremendous promise as therapeutic tools in many diseases. However, the realization of optimal stem cell therapy is critically dependent on the successful retention of implanted cells at the site of injury and their effective incorporation into the damaged tissue. Mesenchymal stem cells (MSC) are a potential source of stem cells that have been shown to be effective in a range of cellular therapies in tissue engineering and regenerative medicine, but the biologic mechanisms underlying their function are just being elucidated. This knowledge is clearly essential to improving and optimizing stem cell therapies going forward.

While no single cell surface marker unequivocally identifies MSCs from all tissues, consensus in the field has proposed three minimal criteria to distinguish MSCs from other hematopoietic stem cells (Dominici et al., 2006). Characteristic MSCs (1) adhere to plastic, (2) express a characteristic pattern of cell surface molecules, and (3) can be differentiated into chondroblasts, adipocytes and osteoblasts *in vitro*. Additional cell surface markers have been identified as being expressed on MSCs, but as they are also expressed on other cells are not always included in the profile. CD13 is a member of this latter group and has been shown to be expressed on embryonic and adult stem cells isolated from numerous sources (Aust et al., 2004; Covas et al., 2005; Fan et al., 2005; Musina et al., 2005; Trubiani et al., 2005; Seeberger et al., 2006). However, potential functional roles for CD13 in these cells have not been investigated.

CD13 is a type II zinc-dependent metallopeptidase (also known as aminopeptidase N) that is found on the surface of all myeloid cells in addition to pericytes, activated endothelial cells, and subsets of organ-specific epithelial cells (Funk et al., 1994; Jamur et al., 2005; Mina-Osorio, 2008; Armulik et al., 2011). It is a multifunctional protein with both enzyme-dependent and independent functions that contribute to adhesion, cell migration, angiogenesis, inflammatory trafficking, adhesion, antigen presentation, and endocytosis (Shipp and Look, 1993; Bhagwat et al., 2003; Luan and Xu, 2007; Petrovic et al., 2007; Winnicka et al., 2010; Ghosh et al., 2012; Pereira et al., 2013; Rahman et al., 2013; Subramani et al., 2013).

In this study, we phenotypically and functionally characterized bone marrow-derived MSC from wild type and CD13^KO^ mice. Isolated cells of both genotypes expressed normal profiles of characteristic stem cell markers and were capable of differentiation into the adipogenic and osteogenic lineages. However, functional analysis showed that CD13 is important for optimal MSC adhesion, migration and vascular network formation. In addition, FAK phosphorylation is diminished and cytoskeletal architecture is disrupted in CD13^KO^ MSCs. Finally, CD13^KO^ MSC were impaired in their ability to mediate the recovery of perfusion in a murine model of hind limb ischemia *in vivo*.

## Materials and methods

### Animals

Global CD13^KO^ mice were generated at the Gene Targeting and Transgenic Facility at the University of Connecticut Health Center (Winnicka et al., 2010) and back-crossed for 10 generations to the FVB strain (The Jackson Laboratory, Bar Harbor, ME). All animals were housed under specific pathogen-free conditions with 12 h light/dark cycle and controlled temperature at the University of Connecticut Health Center animal facilities in accordance with Institutional and Office of Laboratory Animal Welfare guidelines. 7–8 weeks old mice were used for all experiments.

### Bone marrow derived mesenchymal stem cells isolation and culture

MSC were isolated and cultured as previously described (Peister et al., 2004). Briefly, the femurs and tibiae were removed, cleaned, and flushed the marrow cells from 6 to 8 weeks old WT and CD13^KO^ mice. Total mononuclear cells were cultured in DMEM, with 15% FBS on plastic dishes at 37°C in 5% humidified CO_2_. After 24 h., non-adherent cells were washed off and adherent cells were expanded. When adherent cells were confluent (defined as passage 0), they were continuously cultured as MSCs until passage 3. All primary cell experiments used cells at passage 4–7 to avoid both hematopoietic cells contamination and long-term culture effects.

### Human bone marrow mesenchymal stem cell culture

Human MSCs were purchased from Thermo Scientific (#SV30110.02) and cultured with mesenchymal stem cell medium (MSCM) from ScienCell (#7501) that is a complete medium designed for optimal growth of normal human MSCs *in vitro*.

### Reverse transcription PCR analysis

The total cellular RNA was isolated from the wild type MSCs (WT-MSCs) and CD13^KO^ MSCs (KO-MSCs). PCR amplification was performed using Invitrogen Superscript III Reverse Transcriptase and other reagents according to manufacturer's instructions (Invitrogen Corporation, Carlsbad, CA). For PCR, we used primers for Sca1, CD29, CD44, CD49e, and CD105. All of the primer sequences were determined using established GenBank sequences. Duplicate PCR reactions were amplified using primers designed GAPDH as a control for analysis by agarose gel electrophoresis.

### Flow cytometric assessment of cellular infiltration

Flow cytometric analysis was used to characterize the phenotypes of the MSCs. Cells were lifted with trypsin/EDTA and counted. About 1 × 10^5^ cells (in 100 μl PBS/0.5% bovine serum albumin/2 mmol/l EDTA) were incubated with fluorescence-labeled monoclonal antibodies against mouse CD29, CD49e, CD34, CD45, CD11b at 4°C for 30 min. All antibodies were purchased from Biolegend. Flow cytometry was performed on LSRII (Becton Dickinson) and the data analyzed with FlowJo software (Tree Star).

### Adipogenic and osteogenic differentiation

Passage 4 MSCs were incubated to differentiate into adipocytes and osteoblasts in corresponding induction medium for 3 weeks (Peister et al., 2004). For adipogenesis, the cultures were incubated in DMEM that was supplemented with 15%FBS, 100 U/mL penicillin, 100 μg/ml streptomycin, 12 mM L-glutamine, 5 μg/ml insulin (Sigma), 50 μM indomethacin (Sigma), 1 × 10^−6^ M dexamethasone, and 0.5 μM 3-isobutyl-1-methylxanthine (IBMX; Sigma). The medium was changed 2 times per week for 3 weeks. The cells were fixed with 10% formalin for 20 min at RT and stained with 0.5% Oil Red O (Sigma) in methanol (Sigma) for 20 min at RT. For osteogenesis, the cultures were then incubated in DMEM that was supplemented with 15% FBS, 100 U/mL penicillin, 100 μg/ml streptomycin, 12 mM L-glutamine, 20 mM β-glycerol phosphate (Sigma, St Louis, MO), 50 ng/ml thyroxine (Sigma), 1 nM dexamethasone (Sigma), and 0.5 μM ascorbate 2-phosphate (Sigma). The media was changed 2 times per week for 3 weeks. The cells were fixed with 10% formalin for 20 min at RT and stained with Alizarin Red, pH 4.1 (Sigma) for 20 min at RT.

### Adhesion assays

Wells of a 96-well plate (Reacti-bind™, Pierce Biotechnology) were coated with fibronectin (10 μg/ml), Matrigel (1:100 dilution), or 1% gelatin overnight at 4°C, washed, blocked with 100 μl 1% boiled BSA for 1 h at room temperature. Cells (1 × 10^4^ /well/150 μl) were plated for 60 min at 37°C, washed three times, and stained with 0.5% crystal violet for 30 min. Plates were washed six times with PBS, solubilized with 100 μl 1% SDS solution and adhesion was quantified with a spectrophotometer at 595 nm (Mina-Osorio et al., 2008; Kim et al., 2012).

### MTT proliferation assay of MSCs

Cells were plated at a density of 6000 cells/ well/ 200 μl in a 96 well plate (None and Fibronectin coated) and were incubated with complete medium. MTT (20 μl, 5 mg/ml) was added to each well at indicated time points and incubated for 3.5 h. MTT converted in living cells was solubilized with 4 mM HCl, 0.1% Nonidet P-40 (NP40) in isopropanol and absorbance measured at 595/655 nm.

### Cell migration and invasion assays

Cell migration and invasion assays were performed using a BD FluoroBlok 24-multiwell insert system (BD Biosciences). The inserts contain a fluorescence-blocking, 8-μm pore size membrane. The FluoroBlok allow quantification of the number of cells that have migrated through the pores by microscope. To study cell migration, MSCs were suspended in serum-free DMEM medium, and seeded on a BD Falcon FluoroBlok 24-multiwell insert (0.25 ml of cells suspension, 1 × 10^4^ cells per top chamber). To study cell invasion, the FluoroBlok were coated with Matrigel (1:5 dilutions) for 2 h at 37°C. MSCs were suspended in serum-free DMEM medium, and seeded on coated FluoroBlok (0.25 ml of cells suspension, 1 × 10^5^ cells per top chamber). The bottom chambers contained 0.75 ml of 10% FBS contained DMEM medium. Cells were incubated in the FluoroBlok multiwell insert system for 4 h. (migration) and for 6 h (invasion) at 37°C in a humidified atmosphere of 5% CO_2._ Carefully cut the FluoroBlok and coverslipped on slides using Dapi Vectashield mounting medium (Vector Laboratories, Burlingame, CA). Photographs and of migrated cells were taken with Axiocam MRC camera (0.63X magnification) attached to Zeiss Axioplan 2 microscope using a 10x objectives and counted them.

### Endothelial cell network formation assay

WT and CD13^KO^ MSCs (1 × 10^5^) were seeded on Matrigel (BD Biosciences, Bedford, MA) coated 24 well plates with DMEM (10% FBS). After 12 h, images were acquired at 20X magnification (2X objective) using a Nikon T-BPA camera attached to the Nikon Eclipse TE2000-U. The software used was SPOT version 4.1. Three individual experiments were performed. Total numbers of branch points per well were enumerated.

### Western blotting

Isolated primary MSCs were lysed in ice-cold buffer (1% NP40 lysis buffer with protease and phosphatase inhibitors). Equal amount of protein from each group were separated by SDS-PAGE and transferred on to PVDF membrane and incubated with respective primary antibodies; CD13 monoclonal antibody for mouse CD13 (SL-13, custom made by ProMab Biotechnologies, Inc. Richmond, CA); 452 for human CD13 (Dr. Meenhard Herlyn, Philadelphia, PA); pFAK 397, pFAK925, and tFAK (cell signaling); β-Actin (Sigma); followed by incubation with horseradish peroxidase-conjugated secondary antibodies. The antigen-antibody complexes were detected with the use of a chemiluminescence reagent kit (Thermoscientific).

### Histology and immunohistochemistry

Mouse or human MSCs were cultured on slides and fixed in 4% paraformaldehyde solution, permeabilized with 0.2% Triton X-100 for 10 min, and blocked with 5% BSA for 1 h. Cells were incubated with SL-13 (dilution 1/500) for mouse CD13 staining; 452 mAb (dilution 1/250) for human CD13 staining; pFAK397 and pFAK925 (cell signaling) for pFAK staining overnight followed by fluorescence secondary antibody (dilution 1/1000) for 1 h at room temperature. For F-actin staining cells were incubated with TRITC-phalloidin (Sigma-Aldrich, 1/100 dilution) for 1 h. overnight followed by fluorescence secondary antibody (dilution 1/1000) for 1 h at room temperature.

After completing blood flow assessments over 21 days, gastrocnemius muscles were dissected, fixed in 4% paraformaldehyde for 24 h, dehydrated, embedded in paraffin, and sectioned at 7 μm thickness. After deparaffinization and rehydration, antigen retrieval was done with citrate buffer pH 6 and sections blocked and incubated overnight at 4°C with primary antibodies followed by fluorescent secondary antibody for 1 h at room temperature. The capillaries were visualized by immunofluorescent staining with anti-CD31 (Santa Cruz Biotechnology) (dilution 1/200). Respective fluorophore-conjugated secondary antibodies (Molecular Probes, Carlsbad, CA) (dilution 1/1000) were used. The slides were coverslipped using Dapi Vectashield mounting medium (Vector Laboratories, Burlingame, CA). The capillary density is assessed relative to the number of muscle fibers. Muscle regeneration (fibers with centrally located nuclei/total fiber #) in the crural muscle was analyzed by haematoxylin and eosin staining.

Tissue sections were photographed with Optronics camera attached to Ziess Axioskop 2 plus microscope using the Zeiss Achroplan 20X objective and images were captured using MagnaFire SP 2.1B software. Fluorescence images were photographed with Axiocam MRC camera (0.63X magnification) attached to Zeiss Axioplan 2 microscope using a 10x, 20X, 40X or 63X objectives. For fluorescence quantification all of the images were acquired at the same exposure.

### Quantitative cell adhesion assay and CD13 cross-linking

Monolayer adhesion assays were performed as described previously (Mina-Osorio et al., 2008). In brief, human MSCs (1 × 10^5^) were labeled with calcein for 30 min at 37°C followed by treating with activating anti-CD13 452 mAb for 30 min with or without, washed and allowed to adhere to HUVEC monolayer cells for 45 min, lysed and fluorescence read at 485/530 nm and expressed as relative fluorescence unit (RFU).

For cross-linking of CD13 on human MSCs, cells were incubated with control anti-CD13 452 mAb in culture medium for 0, 5, 15, and 30 min at 37°C in a humidified 5% CO2 incubator. Immediately after cross-linking, the reaction was stopped by adding 5 mL of cold PBS and washed once. Cells were lysed in 1.0% NP-40 lysis buffer (20.0 mM HEPES pH 7.4, 150 mM NaCl, and 1.0% NP-40) with protease inhibitor cocktail (Roche) and phosphatase inhibitors. Lysates were cleared by centrifugation at 7000 rpm for 15 min. Proteins or immunoprecipitates were diluted with 4X sample buffer and resolved by 10% SDS-PAGE and electrotransfered onto a polyvinylidene difluoride membrane (Millipore, Bedford, MA) followed by probing with pFAK397 (1:1000), followed by HRP-conjugated secondary Abs (1:5000) and detected using the ECL- kit (Thermoscientific, USA). The blot also stripped for tFAK and β-Actin detection.

### Hindlimb ischemia model and cell transplantation

All animal procedures were performed in accordance with the guidelines approved by the Animal Care Committee of the University of Connecticut. Surgical grade anesthesia was induced by intraperitoneal injection of Ketamine (100 mg/kg) and Xylazine (10 mg/kg). The right femoral artery was ligated proximal to the deep femoral artery and distal to saphenous artery. The deep femoral artery, superficial branches and bifurcation of the popliteal artery were cauterized, and the femoral artery was completely removed between the two ligatures avoiding injury of the femoral vein and nerve to preclude influence of inflammation and edema on arteriogenesis and angiogenesis. Postoperative analgesia was provided with buprenorphine (0.05 mg/kg). After 4 h., the injection of PBS, wild type MSC (WT-MSC), and CD13^KO^ MSC (KO-MSC) were performed intramuscularly by injecting cells (2 × 10^6^) resuspended in PBS at three different points (20 μl in each point) into the gastrocnemius muscles of wild type mice with a 27 g needle (Kim et al., 2012).

### Laser-doppler perfusion imaging

Non-invasive measurements of superficial hindlimb perfusion were obtained before and 0, 3, 7, 14, and 21 days after ligation using a Laser Doppler perfusion imager (model LDI2-IR, Moor Instruments, Wilmington, DE) that was modified for high resolution and depth of penetration (2 mm) with and 830 nm wavelength infrared 2.5 mW laser diode, 100 μm beam diameter, and 15 kHz bandwidth. At each time point, an average of 4 measurements per animal was made on anesthetized (1.5% isofluorane on an isothermal heating pad). To avoid the influence of light and temperature, the results were expressed as a ratio of perfusion in the right (ischemic) vs. left [non-ischemic (NI)] limb (Limbourg et al., 2009).

### *in vivo* assessment of limb function and ischemic damage

Semi quantitative assessment of impaired use of the ischemic limb (ambulation score) was performed using the following criterion: 3 = most severe, unable to use the foot, dragging foot; 2 = no dragging, but no plantar flexion (ability to flex the ankle); 1 = positive plantar flexion; and 0 = able to flex toes to grasp cage in response to gentle traction on the tail (Stabile et al., 2003). Semi quantitative measurement of the ischemic damage (necrosis score) was also assessed (1 to 5 = one to five fingernails damaged, 6 to 10 = one to five fingers fully damaged, 11 = total paw damage).

### Quantification of cell engraftment in ischemic hindlimbs

Cell engraftment in the ischemic hindlimb was quantified by histological analysis. Briefly, red fluorescent dye PKH26 labeled WT-MSC (2 × 10^6^) and green fluorescent dye PKH67 labeled KO-MSC (2 × 10^6^) were injected into ischemic hindlimbs of wild type mice. After 7 days, the ischemic hindlimbs were harvested, and tissue sections were embedded and sectioned. Five fields from four tissue sections were randomly selected, and the number of labeled cells was counted in each field (Kim et al., 2012).

### Statistical analysis

The data were represented as mean ± s.e.m. of the indicated number of measurements. Statistical differences between groups were analyzed by using unpaired, two-tailed *t*-test or One-Way ANOVA. Differences were considered significant at *p* < 0.05.

## Results

### Mesenchymal stem cell culture and characterization

**Figure 1:**
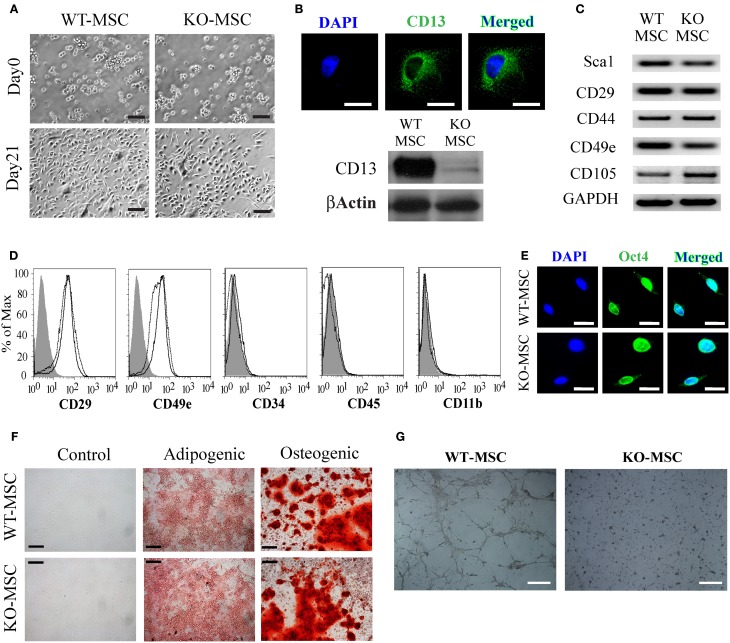
**Mesenchymal stem cell culture and characterization. (A)** Phase contrast image of BM derived MSCs at Day 0 and Day 21(Bar = 100 μm). **(B)** CD13 expression in WT-MSCs by fluorescence immunostaining (Bar = 20 μm) and protein expression of CD13 in WT-MSC. **(C)** RT PCR analysis of stem cell expression profiles. **(D)** Flow cytometric analysis of MSCs. Cells were characteristically positive for CD29, CD49e and negative for CD19, CD31, and CD34. Unstained;WT-MSC; KO-MSC–. **(E)** Both WT-MSC and KO-MSC expressed transcription factor OCT3/4 (Bar = 20 μm). **(F)** Confluent MSCs were transferred to adipogenic and osteogenic medium for 3 weeks. Adipocytes were detected by oil red O staining and osteoblasts by alizarin red staining (Bar = 200 μm). **(G)** 1 × 10^5^ cells were seeded on Matrigel coated 6-well plates and incubated for 12 h. cells isolated from CD13^KO^ mice are unable to form capillary networks and form fewer branches (Bar = 200 μm).

To determine if CD13 contributes to the biologic function of stem cells we isolated MSCs from the bone marrow of wild type and CD13^KO^ mice. Cells of both genotypes were grossly visually similar upon isolation and throughout the experimental culture period (Figure 1A) and as expected, the CD13 protein was abundantly expressed on wild type but not CD13^KO^ MSCs (Figure 1B). RT-PCR and flow cytometric analyses illustrated that cultured cells of both genotypes expressed equivalent levels of the characteristic cell surface MSC markers (Figures 1C,D). Similarly, immunofluorescent staining for the pluripotency marker Oct4 verified the multipotent potential of both wild type and CD13^KO^ MSCs (Figure 1E). Furthermore, characterization of cultured wild type and CD13^KO^ MSCs showed comparable capacities to form adipocytes and osteoclasts under conditions reported to induce adipogenic and osteogenic differentiation (Figure 1F). Interestingly, and consistent with our previous data implicating CD13 as a functional regulator of angiogenesis (Pasqualini et al., 2000; Bhagwat et al., 2001, 2003; Petrovic et al., 2007) CD13^KO^ MSCs were incapable of forming endothelial networks (Figure 1G). These results confirmed CD13 as a MSC marker and suggest that CD13 is not necessary for the formation of MSC in the bone marrow or their short-term survival *in vitro* after isolation. However, CD13 is required for MSC to differentiate toward some but not all cell lineages.

### CD13^KO^ mesenchymal stem cells are functionally impaired

**Figure 2:**
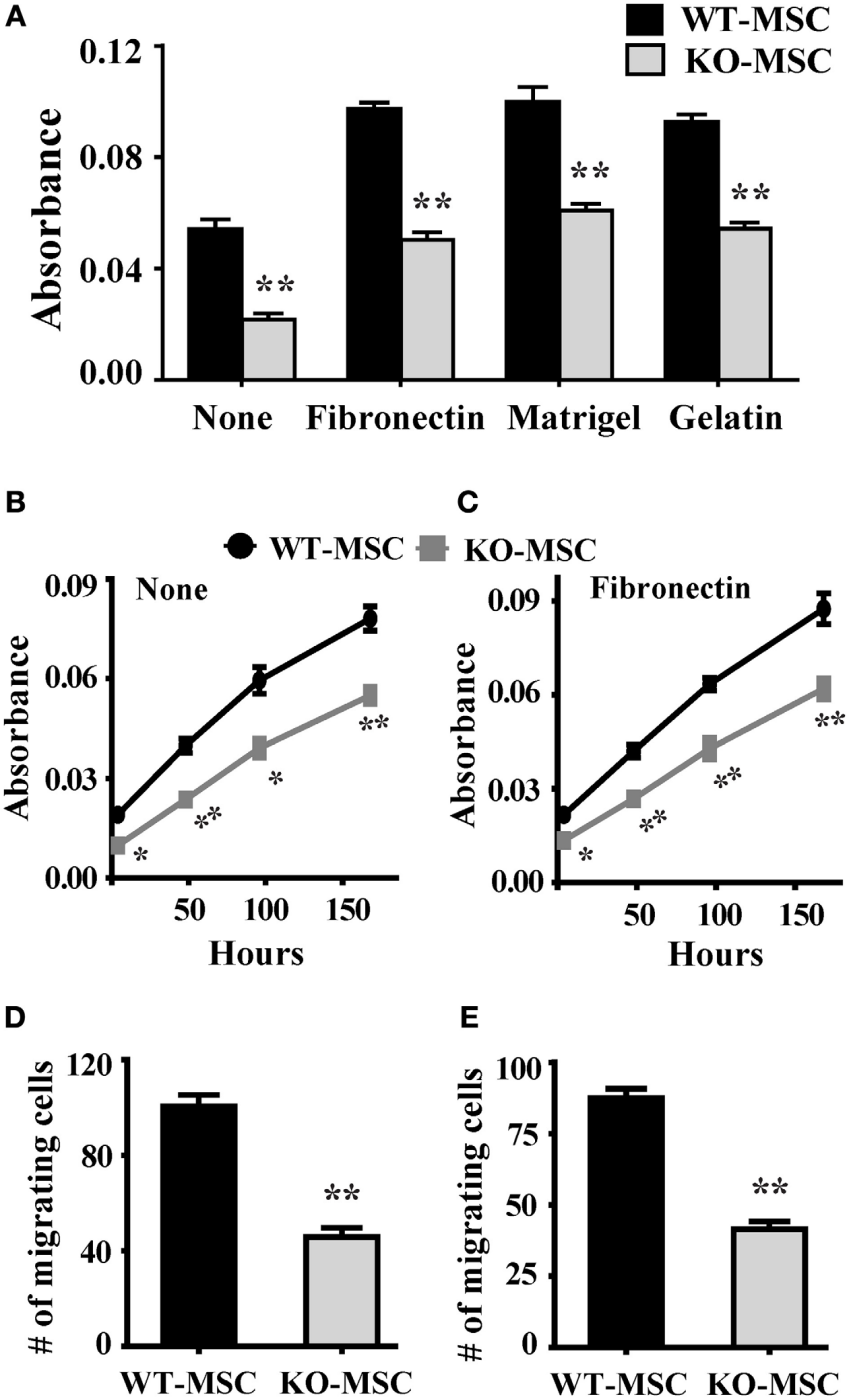
**Lack of CD13 impairs MSC adhesion, proliferation, migration, and invasion. (A)** Adhesion assay: Cells (1 × 10^4^) were seeded in 96 well plates coated separately with fibronectin, Matrigel, or gelatin and allowed to adhere for 1 h at 37°C. After PBS wash, adherent cells were detected by MTT assay. *n* = 6, ^**^P < 0.01. **(B,C)** Proliferation assay: Cells (0.5 × 10^4^) were seeded in 96 well plate and cell proliferation detected by MTT assay at the indicated time points. *n* = 6, ^*^P < 0.05, ^**^P < 0.01. **(D)** Migration assay: 1 × 10^4^ cells were seeded in FluoroBlok chambers. After 4 h. incubation the cells were stained with DAPI and counted. *n* = 4, ^**^P < 0.01. **(E)** Invasion assay: 1 × 10^5^ cells were seeded on Matrigel (1:5 dilution) coated FluoroBlok chambers. After 6 h. incubation the cells were counted. *n* = 4, ^**^P < 0.01.

We have previously demonstrated that CD13 functions as an adhesion molecule regulating monocyte-endothelial interactions (Mina-Osorio et al., 2008; Subramani et al., 2013) and is required for endothelial cell invasion (Bhagwat et al., 2003; Petrovic et al., 2007). To determine if CD13 functioned similarly in MSCs we tested wild type and CD13^KO^ MSCs in *in vitro* in adhesion, migration and invasion assays; functions that are dependent on adhesion. Assessment of MSC adhesion to matrix proteins contained in preparations of human fibronectin, Matrigel or gelatin (Figure 2A) indicated that CD13^KO^ MSCs are significantly less adherent than wild type cells to all ECM proteins tested, suggesting that this effect is not strictly matrix or ligand dependent and that CD13 plays a more universal role in cell attachment. In contrast, proliferation rates as measured by the MTT assay are similar over time (Figures 2B,C), although lower initial absorbance readings for CD13^KO^ cells in this assay likely reflect their overall reduced adherence. Similar to our results in endothelial cells, both migration and invasion of MSC toward chemotactic stimuli were impaired (Figures 2D,E). Therefore, lack of CD13 hinders MSC functions *in vitro*, consistent with a loss in adhesive properties.

### Adhesion-regulating signal transduction pathways are altered in CD13^KO^ MSCs

**Figure 3:**
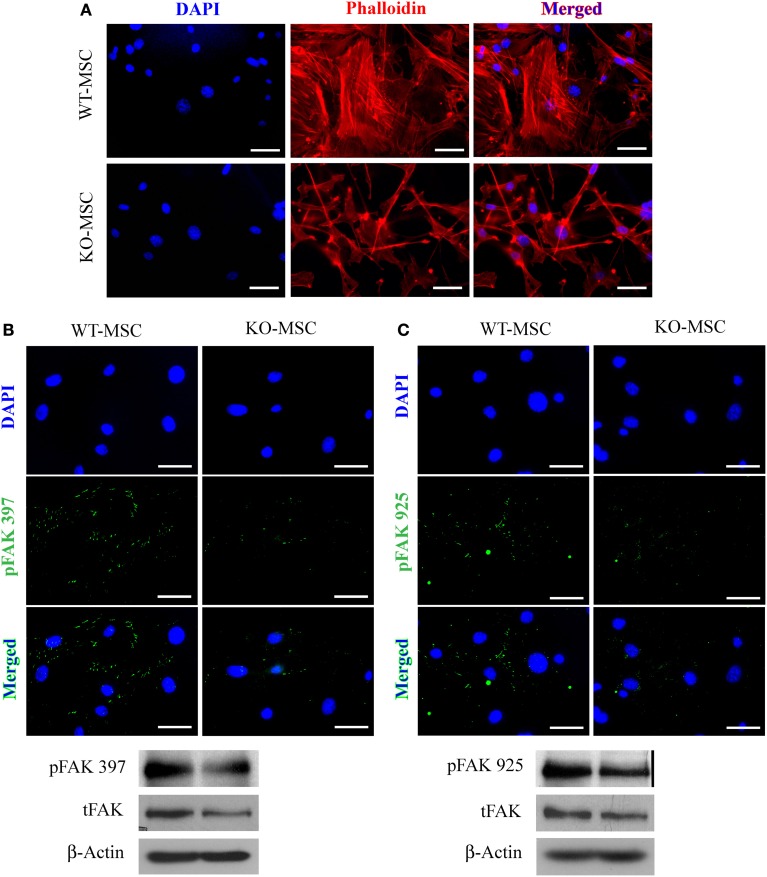
**Mesenchymal stem cell defects in CD13^KO^ mice are cell intrinsic. (A)** phalloidin-stained MSC cells isolated from injured muscles of CD13^KO^ mice showed remarkable cytoskeletal disruption compared to cells from WT mice; Objective 40X (Bar = 100 μm). **(B,C)** Immunofluorescent detection of FAK phosphorylation at tyrosine residues 397 **(B)** and 925 **(C)**. Protein lysates of MSC were probed for phospho-FAK (Y397) and phospho-FAK (Y925) with β-actin as the loading control. CD13^KO^ MSCs expressed lower levels of phospho-FAK protein (Bar = 20 μm).

Adhesion to the extracellular matrix via adhesion molecules activates well-characterized signal transduction cascades that induce intracellular alterations in the cytoskeleton. Staining for intracellular F-actin in wild type and CD13^KO^ MSCs with phalloidin shows an obvious disruption of cytoskeletal integrity in the absence of CD13 (Figure 3A). Accordingly, dramatic reductions in phosphorylation of FAK at residues 397 and 925 and a nearly complete absence of focal adhesions clearly indicate severely disordered adhesion processes, although total FAK protein levels are unchanged (Figures 3B,C) suggesting CD13 regulates MSC adhesion via FAK activation as we have previously shown in monocytes (Subramani et al., 2013).

### CD13 activation induces adhesion and FAK activation in human MSCs

**Figure 4:**
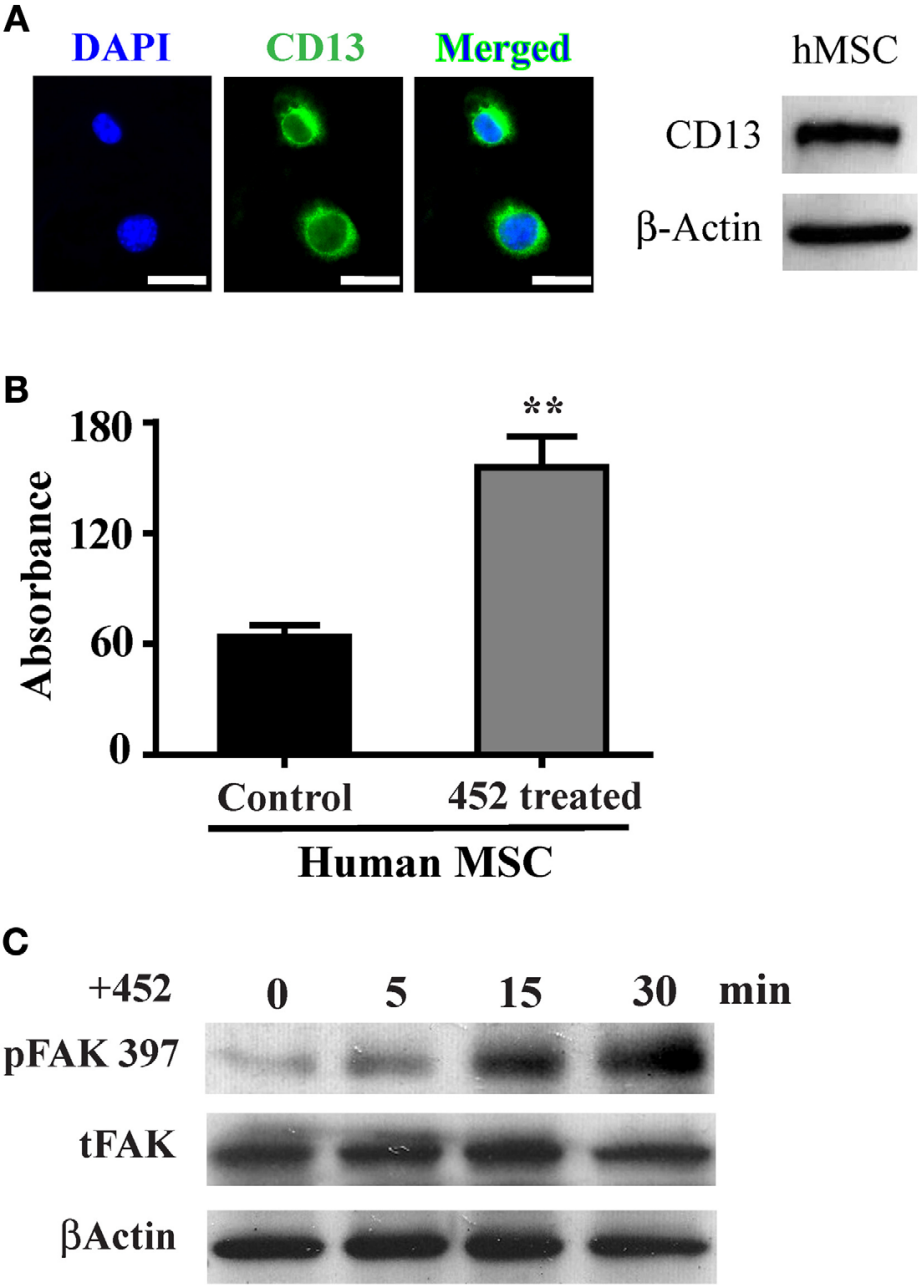
**CD13 activation increases monolayer adhesion and FAK phosphorylation in human MSCs. (A)** Human mesenchymal stem cells also express CD13 by immunofluorescence (green); Objective 63X (Bar = 20 μm) and immunoblot of human cell lysates. **(B)** Colorimetric quantification of adhesion of human MSCs treated with the CD13 activating mAb 452 to HUVEC monolayers. Data represents the mean ± s.e.m. *n* = 3 from two independent experiments (^**^P < 0.01). **(C)** CD13 crosslinking with activating mAb 452 temporally induces FAK tyrosine phosphorylation in human MSCs.

We have demonstrated that activation of CD13 with a ligand mimicking monoclonal antibody increases monocyte adhesion to endothelial cells (Mina-Osorio et al., 2008; Subramani et al., 2013). CD13 is also prominently expressed in human MSCs (hMSC, Figure 4A) and may similarly mediate MSC-endothelial adhesion. Crosslinking of hMSC CD13 with the activating mAb 452 induces cell-cell adhesion (Figure 4B) and FAK phosphorylation (Figure 4C), suggesting that CD13 can function as a signal transducing adhesion molecule to mediate MSC adhesion, migration and invasion.

### CD13^KO^ mesenchymal stem cells are impaired in enhancing wound healing *in vivo*

**Figure 5:**
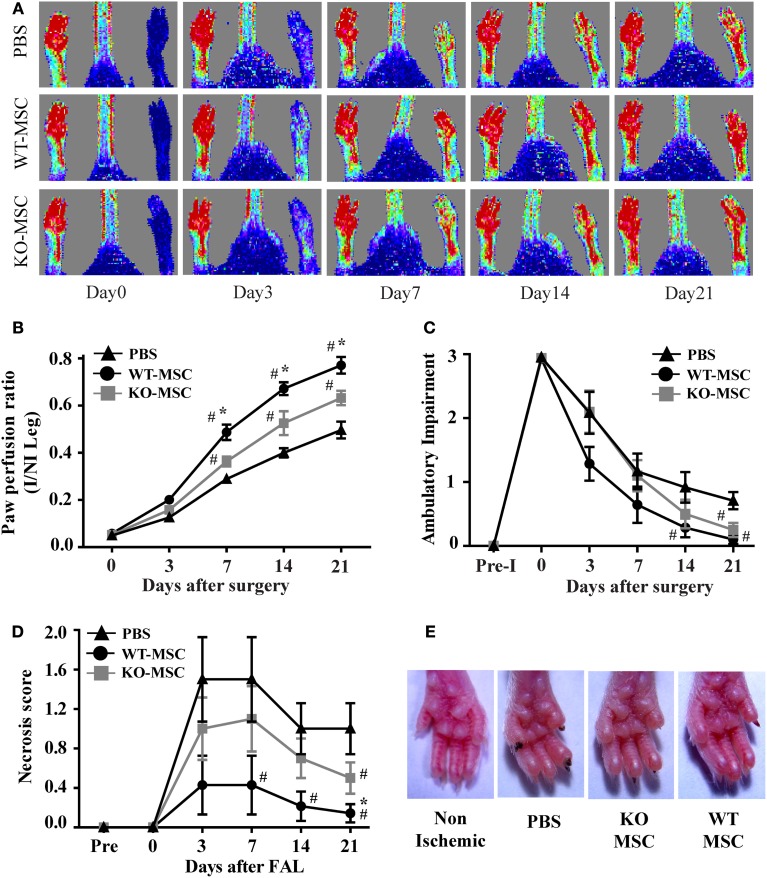
**Lack of CD13 on exogenously administered MSCs impairs perfusion recovery in an *in vivo* hindlimb ischemia model. (A)** Laser Doppler flow imaging of perfusion in mice at the indicated time points. Representative color-coded images of three groups (PBS, WT-MSC, and KO-MSC) of mice on day 0, 3, 7, 14, and 21 after surgery and cell transplantation assessed by laser Doppler imaging. Red indicates highest perfusion velocity, green intermediate, and blue, lowest velocity. **(B)** Cumulative results for PBS (*n* = 7), WT-MSC (*n* = 6), and KO-MSC (*n* = 6) injected mice are shown graphically as ratios of blood flow in ischemic limb (I) to that in the non-ischemic limb (NI) at each time point. Functional assessment of ischemic muscles. Cumulative results are shown graphically as **(C)** the ambulatory impairment score; **(D)** ischemic tissue damage score as described in Methods. **(E)** Images of Non-ischemic and PBS, WT-MSC, and KO-MSC injected ischemic paw. Black nail indicates necrosis. ^#^*P* < 0.05 compare to PBS and ^*^P < 0.05 compare to KO-MSC; (score assessment criteria in Methods).

It is well established that administration of exogenous MSCs substantially contributes to wound repair in the hind-limb ischemia model of angiogenesis. To assess the effect of the lack of CD13 in MSC function *in vivo*, we removed the femoral artery and collateral vessels from single flanks of WT mice and injected randomized animals either with purified WT or CD13^KO^ MSCs or PBS control into the surgery site. Laser Dopplar imaging of blood flow immediately following ligation clearly showed that the ligated leg is poorly perfused (blue color) relative to the contralateral leg (Figure 5A, day 0), but that circulation is progressively re-established over a period of 3 weeks (Figure 5A). In agreement with published studies, this revascularization is significantly improved in the animals injected with wild type MSCs compared to animals injected with vehicle control (Figure 5B). In contrast, injection of CD13^KO^ MSCs showed a significant and prolonged delay in recovery of blood flow over 21 days post-injury, suggesting that impaired MSC adhesion *in vitro* predicts reduced MSC function *in vivo* in the absence of CD13. In agreement with this result, we found reduced ambulatory capacity (impaired limb function, Figure 5C) and a higher degree of paw necrosis in the CD13^KO^ animals (Figures 5D,E).

### Muscle generation and capillary formation are impaired in mice injected with CD13^KO^ MSC following ischemic injury *in vivo*

**Figure 6:**
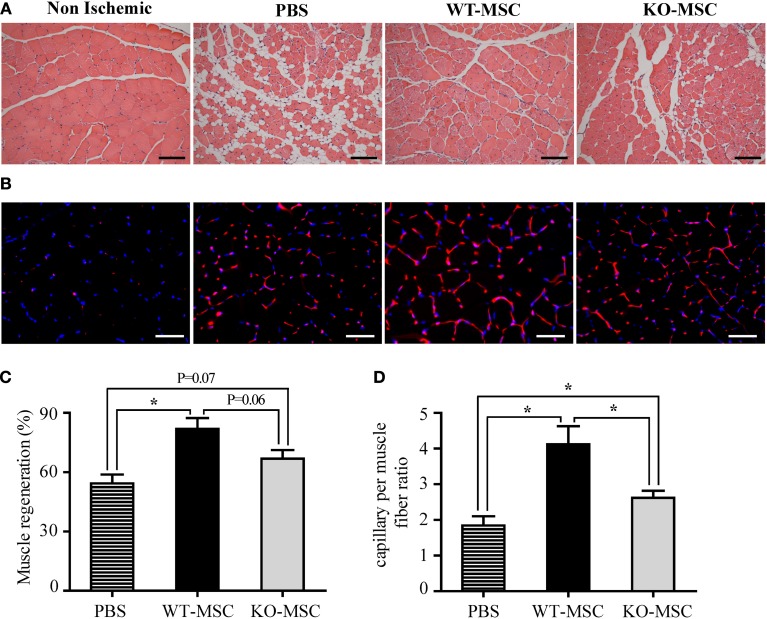
**Muscle regeneration and capillary formation are impaired in mice injected with CD13^KO^ MSC following ischemic injury. (A)** Hematoxylin and eosin (H&E) staining of gastrocnemius muscle regeneration was confirmed by the presence of multiple, centrally located myocyte nuclei, 20X objective; Bar = 100 μm. **(B)** Capillaries were visualized by immunofluorescent staining with CD31 (red) and nuclei with DAPI (blue); Objective, 40X objective; Bar = 50 μm. **(C)** The area of the injured tissue was compared among PBS, WT-MSC, and KO-MSC groups. A significant increase in muscle regeneration (average) was observed in WT-MSC group compared with PBS group at day 21. **(D)** The ratio of capillary density per fiber was measured in ischemic gastrocnemius muscles. Capillary density was significantly increased in WT-MSC compared with other groups, PBS and KO-MSC. All data were quantified by ImagePro Plus. Values are shown as mean ± s.e.m. (^*^P < 0.05) (PBS *n* = 7; WT-MSC *n* = 6; KO-MSC *n* = 6).

Histologic analysis of muscles from animals receiving WT MSCs at 21 days post-surgery/injection showed clear evidence of regenerating muscle as illustrated by numerous myofibers with centrally located nuclei (WT- Figure 6A) where vehicle controls showed marked metaplasia with loss of myofibers and decreased muscle regeneration characteristic of impaired muscle recovery Figure 6A, PBS (Limbourg et al., 2009). While CD13^KO^ MSCs contribute to healing, muscle recovery is noticeably reduced (Figure 6A, KO and Figure 6C). Similarly, femoral artery removal results in hypoxia that triggers a robust angiogenic response and exogenously administered MSCs can enhance this revascularization of injured tissue (Limbourg et al., 2009). Immunofluorescent analysis of the vascular response to injury indicate that the density of CD31+ endothelial cell-lined luminal capillaries is significantly decreased in muscles of mice receiving CD13^KO^ MSCs (Figures 6B,D). In addition, these structures appeared more immature with fewer characteristic branches in recipients of CD13^KO^ MSCs, confirming our *in vitro* observations that CD13 is required for angiogenesis and suggesting that CD13 promotes MSC-mediated wound healing and revascularization in this model of ischemic injury.

### CD13 prolongs the survival and engraftment of mesenchymal stem cells

**Figure 7:**
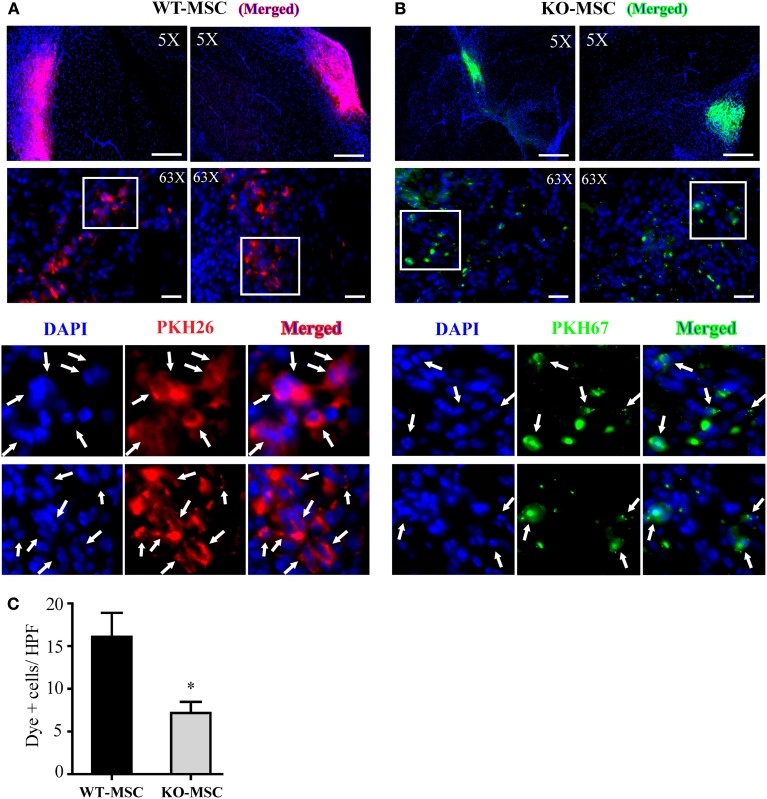
**Engraftment of MSCs *in vivo*.** Representative images of localized PKH26 (red) labeled WT-MSCs **(A)** and PKH67 (green) labeled KO-MSCs **(B)** at 7 days after cell injection. **(C)** Quantification of engrafted MSCs. Dye positive cells was quantified. ^*^P < 0.05 WT-MSC vs. KO-MSC. 5X objective, Bar = 500 μm; 63X objective, Bar = 50 μm.

The impaired muscle regeneration in recipients of CD13^KO^ MSCs suggests that CD13-dependent adhesion is important for the engraftment of MSC. To estimate the relative engraftment potential of WT and CD13^KO^ MSCs in ischemic hindlimbs, we directly transplanted a total of 2 × 10^6^ differentially PKH dye-labeled cells of each genotype into the ischemic region of the hindlimb injury. 7 days post-injury/transplantation, hindlimb tissues were collected and analyzed for the number of dye labeled cells remaining in the wound by fluorescence microscopy (Figures 7A,B). Significantly higher numbers of transplanted WT MSCs were detected in tissues (PKH+/nuclear-DAPI+) than CD13^KO^ MSCs (Figure 7C), suggesting that CD13 regulates the function of transplanted MSCs in the wound; potentially at the level of retention, survival, or engraftment potential, thus, significantly contributing to the ability of exogenous MSCs to facilitate wound healing in ischemic injury.

## Discussion

CD13 was originally identified as a marker of myeloid leukemia and normal hematopoietic cells of the myeloid lineage (Subcomittee, 1984) and was subsequently discovered to be identical to the cell surface peptidase Aminopeptidase N (Look et al., 1989). Further studies by our group and others have identified numerous functional roles for this molecule in various tissues in addition to hematopoietic cells, some of which are enzyme-dependent [cleavage of bioactive peptides (Gros et al., 1985), reabsorption of amino acids (McClellan and Garner, 1980)] and others that are independent of its enzymatic activity [viral receptor (Yeager et al., 1992), adhesion molecule (Mina-Osorio et al., 2008), endocytic mediator (Ghosh et al., 2012) and angiogenic regulator (Bhagwat et al., 2001)]. The prominent and pervasive expression of CD13 on embryonic and adult stem cells of numerous origins (Aust et al., 2004; Covas et al., 2005; Fan et al., 2005; Musina et al., 2005; Trubiani et al., 2005; Seeberger et al., 2006) prompted the current investigation into possible roles for this multifunctional molecule on MSC biology. Comparison of the *in vitro* and *in vivo* properties of bone marrow-derived mesenchymal stem cell populations isolated from wild type and CD13^KO^ mice showed that MSC-expressed CD13 serves many of the functions that have been demonstrated on other cells and that lack of CD13 on MSCs has profound effects on the ability of exogenously administered cells to contribute to healing of skeletal muscle following severe ischemic injury.

Initial characterization of our global CD13^KO^ animals showed that unchallenged mice are healthy and fertile with essentially normal hematopoietic profiles and physiologic myeloid functions (Winnicka et al., 2010), similar to the normal expression profiles of stem cell markers and proliferation rates we observed in freshly isolated bone marrow derived MSCs. Interestingly, although both wild type and CD13^KO^ MSCs express the pluripotency marker Oct4 and can differentiate into cells characteristic of the osteogenic and adipogenic lineages, MSCs lacking CD13 are unable to form vascular networks *in vitro*. This finding is in agreement our previous work demonstrating that CD13 regulates angiogenesis by transducing signals important to the formation of endothelial filopodia (Petrovic et al., 2007), but also raises the intriguing possibility that CD13 may specify or determine endothelial cell fate. Consistent with this notion, we have shown that transcription factors that mediate CD13 expression in myeloid cells also direct the differentiation of myeloid progenitor cells to macrophages (Hegde et al., 1998, 1999), suggesting that CD13 may also be involved in mechanisms that program the differentiation of specific cell lineages. Studies investigating this interesting possibility are ongoing in our laboratory.

We have also shown that CD13 is a homotypic adhesion molecule that mediates inflammatory interactions between monocytes and endothelial cells and activation of CD13 induces signal transduction, cytoskeletal reorganization and increased adhesion to regulate inflammatory monocyte trafficking (Mina-Osorio et al., 2008; Subramani et al., 2013). In the current study, we demonstrate that phosphorylation of the critical focal adhesion kinase FAK and subsequent adhesion of CD13^KO^ MSCs to the extracellular matrix is also significantly reduced. FAK phosphorylation/activation regulates adhesion, which is fundamental to the processes of MSC survival, migration and invasion that control the ability of MSCs to integrate, survive and contribute to healing at the site of injury (Song et al., 2007; Hu et al., 2011; Liao et al., 2012; Meng et al., 2013). Therefore, the reduced adhesive capacity of MSCs resulting from the loss of CD13 profoundly affects essential, cell intrinsic functions and likely forms the basis of the diminished muscle regeneration seen upon CD13^KO^ MSC treatment.

In keeping with the defective CD13^KO^ MSC morphogenesis and capillary network formation, we also find that capillary density is decreased and the capillaries that are formed are immature and poorly branched in the injuries of recipients of the CD13^KO^ MSCs, clearly contributing to reduced functional recovery. In support of this notion, we have found that angiogenesis is universally impaired in CD13^KO^ animals subjected to ischemic injury models (Pereira et al., 2013; Rahman et al., 2013) or tumors (Pasqualini et al., 2000; Bhagwat et al., 2003). Alternatively, the implanted MSCs have been described as a minor source of healthy precursor cells and wild type tissue-resident endothelial precursors are critical for neovessel formation in the wound. This data would argue that the decrease in angiogenesis and lessened regeneration in the CD13^KO^ may be due to reduced survival or retention of the MSCs in the wound, as these are a rich source of paracrine factors that serve to enhance repair by endogenous cells (Williams and Hare, 2011). Indeed, studies in cardiac cell therapy suggest that only a fraction of the donor cells actually integrate long-term, but rather function more short-term by secreting cytokines that stimulate differentiation of tissue-resident precursors, inhibit fibrosis, increase survival, suppress inflammation, and promote angiogenesis (Schulman and Hare, 2012). We find that by 7 days post-injection, ischemic muscles receiving CD13^KO^ MSCs contained significantly fewer labeled cells than recipients of wild type MSCs, which is consistent with reduced survival or retention of cells lacking CD13 at the site of injection. Interestingly, our observation that activation of CD13 on MSCs induces their adhesion suggests that activation of CD13 may be a mechanism to enhance the adhesion of implanted MSCs to improve integration, paracrine secretion, revascularization, muscle regeneration, and perfusion recovery.

Collectively, our results clearly indicate that CD13 plays a protective role in MSC-mediated skeletal muscle repair, which we believe is primarily due to defects in MSC adhesion and angiogenesis. The multifunctional nature of this molecule is consistent with CD13's regulation of multiple aspects of healing following ischemic injury in the muscle and may be a potentially viable target to improve MSC therapy.

## Author contributions

Developed study concept: M. Mamunur Rahman, Linda H. Shapiro, Jiyeon K. Denninger, Kotaro Takeda, Guo-Hua Fong, Morgan E. Carlson. Designed experiments: M. Mamunur Rahman, Jiyeon K. Denninger, Linda H. Shapiro, Morgan E. Carlson. Performed experiments: M. Mamunur Rahman, Mallika Ghosh, Jaganathan Subramani. Interpreted data: M. Mamunur Rahman, Linda H. Shapiro, Morgan E. Carlson, Mallika Ghosh, Jaganathan Subramani. Wrote manuscript: M. Mamunur Rahman, Linda H. Shapiro, Morgan E. Carlson.

### Conflict of interest statement

The authors declare that the research was conducted in the absence of any commercial or financial relationships that could be construed as a potential conflict of interest.
